# Identification and Functional Characterization of Small Alarmone Synthetases in *Corynebacterium glutamicum*

**DOI:** 10.3389/fmicb.2017.01601

**Published:** 2017-08-21

**Authors:** Matthias Ruwe, Jörn Kalinowski, Marcus Persicke

**Affiliations:** Microbial Genomics and Biotechnology, Center for Biotechnology, Bielefeld University Bielefeld, Germany

**Keywords:** stringent response, (p)ppGpp, alarmone, ActRel, RelS, RelP, phylogeny, pGpp

## Abstract

The hyperphosphorylated guanosine derivatives ppGpp and pppGpp represent global regulators of the bacterial stress response, as they act as central elements of the stringent response system. Although it was assumed that both, (p)ppGpp synthesis and hydrolysis, are catalyzed by one bifunctional RSH-protein in the actinobacterial model organism *Corynebacterium glutamicum* ATCC 13032, two putative short alarmone synthetases (SASs) were identified by bioinformatic analyses. The predicted sequences of both enzymes, designated as RelP^*^_Cg_ and RelS_Cg_, exhibit high similarities to the conserved (p)ppGpp synthetase catalytic domain. In the context of sequence analysis, significant differences were found between the RelP variants of different *C. glutamicum* isolates. In contrast to the bifunctional RelA/SpoT homolog (RSH) protein Rel_Cg_, whose gene deletion results in a reduced growth rate, no change in growth characteristics were observed for deletion mutants of the putative SAS proteins under standard growth conditions. The growth deficit of the Δ*rel* strain could be restored by the additional deletion of the gene encoding RelS_Cg_, which clearly indicates a functional relationship between both enzymes. The predicted pyrophosphokinase activity of RelS_Cg_ was demonstrated by means of genetic complementation of an *Escherichia coli* Δ*relA*Δ*spoT* strain. For the expression of RelP^*^_Cg_, as well as the slightly differing variant RelP_Cg_ from *C. glutamicum* AS1.542, no complementation was observed, concluding that both RelP versions possess no significant pyrophosphokinase activity *in vivo*. The results were confirmed by *in vitro* characterization of the corresponding proteins. In the course of this investigation, the additional conversion of GMP to pGpp was determined for the enzyme RelS_Cg_. Since the SAS species analyzed extend both the network of stringent response related enzymes and the number of substances involved, the study of this class of enzymes is an important component in understanding the bacterial stress response. In addition to the comprehension of important biological processes, such as growth rate regulation and the survival of pathogenic species in the host organism, SAS enzymes can be used to produce novel hyperphosphorylated nucleotide species, such as pGpp.

## Introduction

In order to be able to exist in a natural habitat under fluctuating environmental conditions, bacteria have developed various mechanisms to react appropriately to stress conditions. The highly conserved reaction to nutrient deficiencies in bacteria (Potrykus and Cashel, 2008; Gaca et al., 2015a; Liu et al., 2015) and plant chloroplasts (van der Biezen et al., 2000; Masuda et al., 2008) is referred to as stringent response and characterized by the production of guanosine tetraphosphate (ppGpp) and guanosine pentaphosphate (pppGpp). These hyperphosphorylated guanosine derivatives were first identified in 1969 by Cachel and Gallant by thin-layer chromatography as magic spots 1 and 2 (Cashel and Gallant, 1969), which were later revealed to be ppGpp and pppGpp, also known as alarmones and denoted as (p)ppGpp (Cashel and Kalbacher, 1970; Haseltine et al., 1972). An increase in cellular (p)ppGpp concentration generally results in inhibition of growth as well as global modulation of gene expression (Potrykus and Cashel, 2008). Since (p)ppGpp affects expression or activity of various enzymes that are associated with transcription, translation, DNA replication, and nucleotide production, the stringent response is counted among the global cellular regulation systems (Artsimovitch et al., 2004; Milon et al., 2006; Wang et al., 2007; Tozawa and Nomura, 2011).

At the level of transcription, the accumulation of (p)ppGpp leads to a repression of growth-associated genes, rRNA, and ribosomal proteins as well as to an activation of survival-associated stress responses, nutrient uptake, and amino acid biosynthesis (Gaca et al., 2015a). In addition, (p)ppGpp acts as an inhibitor of a wide range of proteins, such as DNA primase, translation factors, and enzymes of the lipid and nucleotide metabolism (Kanjee et al., 2012). In summary, the stringent response changes the cellular resource distribution toward a state of persistence that allows survival under suboptimal living conditions (Dalebroux and Swanson, 2012; Gaca et al., 2015a).

In principle, the synthesis of (p)ppGpp is performed by pyrophosphorylation of GDP or GTP. Due to their homology to the well-characterized *Escherichia coli* enzymes, the involved pyrophosphokinases are referred to as RelA/SpoT homolog (RSH) enzymes, which catalyze the transfer of pyrophosphate from ATP to the ribose 3′-OH of GDP or GTP (Potrykus and Cashel, 2008). In addition to the synthetase domain, RSH proteins contain an N-terminal pyrophosphohydrolase domain and also possess several small domains in the C-terminal part. These regions, classified as TGS and ACT domains (Wolf et al., 1999; Chipman and Shaanan, 2001), are probably involved in the regulation of the RSH major activities, but their function as well as the regulation in general are not yet fully understood.

The constellation of two multi domain RSH enzymes of which RelA exclusively contains synthetic activity and SpoT shows both, a weak pyrophosphokinase activity and a strong (p)ppGpp hydrolase activity, is a special case existing in *E. coli* and other Gamma- or Betaproteobacteria (Hauryliuk et al., 2015). By contrast, most other bacteria and plants have only a single copy of the RSH gene, encoding a bifunctional enzyme (Potrykus and Cashel, 2008).

In addition to RSH proteins, which contain several domains, single-domain fragments have been discovered by bioinformatic approaches. These include monofunctional (p)ppGpp synthetases, so-called small alarmone synthetases (SASs), which were first discovered in *Streptococcus mutans* and *Bacillus subtilis*, (Lemos et al., 2007; Nanamiya et al., 2008) as well as single-domain (p)ppGpp hydrolases, also termed small alarmone hydrolases (SAHs). For some analyzed SAS enzymes there are indications for transcriptional regulation in the context of stress reactions, such as the presence of numerous antibiotics (D'Elia et al., 2009). However, the exact functional relationships in the context of the stringent response are limited (Potrykus and Cashel, 2008). Using 1,000 genomes in a bioinformatic survey for proteins possessing stringent response-associated sequence motifs and subsequent phylogenetic analysis of the identified candidates, Atkinson et al. proposed a classification of the RSH superfamily in 30 different subgroups, containing 12 classes of SASs and 7 classes of SAHs (Atkinson et al., 2011).

For the industrially relevant production organism *Corynebacterium glutamicum*, it was so far assumed that the (p)ppGpp-associated metabolism is relatively simple. The bifunctional RSH enzyme Rel was proposed to be the central element of (p)ppGpp metabolism, acting as both synthetase and hydrolase (Wehmeier et al., 1998; Brockmann-Gretza and Kalinowski, 2006). Still, the publication of the *C. glutamicum* ATCC13032 genome sequence in 2003 (Kalinowski et al., 2003), as well as the development of increasingly powerful molecular biological and bioinformatic methods provide new insights into the complex system of stringent control. The reinterpretation of previous results and novel findings for different model organisms point to the participation of further enzymes and thus a much more complex (p)ppGpp metabolism.

There are even concerns that (p)ppGpp is not the sole effector of stringent response. In the context of the analysis of the SAS enzyme RelQ from *Enterococcus faecalis*, the importance of a further alarmone species has recently been demonstrated (Gaca et al., 2015b). In addition to GDP and GTP, RelQ_Ef_ can also use GMP as the substrate and thereby produces GMP 3′diphosphate (pGpp). For this substance, which had also been detected in different strains of Actinobacteria decades ago (Oki et al., 1976), regulatory effects have been demonstrated for the first time by Gaca et al. (2015b). Thus, analogous to ppGpp and pppGpp, pGpp directly influences the activity of GTP biosynthesis enzymes and inhibits the *E. coli* RNA polymerase transcription of the *rrnB* P1 promoter.

In this study, we investigate *C. glutamicum* using bioinformatics, molecular biology and biochemical methods with regard to a possible extension of the known (p)ppGpp network, aiming at increasing the knowledge about the important system of stringent response. By investigating different *C. glutamicum* deletion mutants it has been shown that the already known growth defect of a *C. glutamicum* Δ*rel* strain can be compensated by additional deletion of a gene encoding a putative SAS enzyme. The predicted pyrophosphokinase activity of this protein was confirmed both by the complementation of an *E. coli* (p)ppGpp^0^ strain and by the direct detection of different alarmone species in pyrophosphokinase enzyme assays with subsequent LC-MS evaluation.

## Materials and methods

### Bacterial strains and growth conditions

Bacterial strains used in this study are listed in Table 1, plasmids are listed in Table 2.

**Table 1:** Strains used in this study.

**Strain**	**Characteristics or genotype**	**Source or reference**
*C. glutamicum*		
CR099	*C. glutamicum* ATCC 13032, ΔCGP1, ΔCGP2, ΔCGP3, ΔISCg1, ΔISCg2	Baumgart et al., 2013; Unthan et al., 2015
CR099 Δ*rel*	*C. glutamicum* CR099 Δ*rel*	This study
CR099 ΔrelP^*^	*C. glutamicum* CR099 ΔrelP^*^	This study
CR099 Δ*relS*	*C. glutamicum* CR099 Δ*relS*	This study
CR099 Δ*rel*ΔrelP^*^	*C. glutamicum* CR099 Δ*rel*ΔrelP^*^	This study
CR099 Δ*rel*Δ*relS*	*C. glutamicum* CR099 Δ*rel*Δ*relS*	This study
CR099 Δ*rel*ΔrelP^*^Δ*relS*	*C. glutamicum* CR099 Δ*rel*ΔrelP^*^Δ*relS*	This study
*E. coli*		
ER2566	*E. coli* expression strain with a chromosomal copy of the T7 RNA polymerase gene under the control of the lac promoter	New England Biolabs
MG1655	Wild-type *E. coli* MG1655, derived from *E. coli* K12	Blattner et al., 1997
MG1655 Δ*relA*	*E. coli* MG1655 *ΔrelA*	This study
MG1655 Δ*relA*, Δ*spoT*	*E. coli* MG1655 *ΔrelA, ΔspoT*	This study

**Table 2:** Plasmids used in this study.

**Plasmid**	**Characteristics**	**Source or reference**
pBAD24	Arabinose inducible expression vector	Guzman et al., 1995
pBAD::*rel*_Cg_	pBAD24 carrying the gene *rel* (*cg1861*) from *C. glutamicum* CR099	This study
pBAD::relP^*^_Cg_	pBAD24 carrying the gene relP^*^ (*cg1330*) from *C. glutamicum* CR099	This study
pBAD::*relP*_Cg_	pBAD24 carrying the gene *relP* from *C. glutamicum* AS1.542	This study
pBAD::*relS*_Cg_	pBAD24 carrying the gene *relS* (*cg2324*) from *C. glutamicum* CR099	This study
pK18*mobsacB*	Suicide vector for gene deletion by homologous recombination	Schäfer et al., 1994
pK18*mobsacB*_*rel*_Cg_	pK18*mobsacB* carrying the up- and downstream region (500 bp respectively) of the gene *rel* from *C. glutamicum* CR099	This study
pK18*mobsacB*_relP^*^_Cg_	pK18*mobsacB* carrying the up- and downstream region (500 bp respectively) of the gene relP^*^ from *C. glutamicum* CR099	This study
pK18*mobsacB*_*relS*_Cg_	pK18*mobsacB* carrying the up- and downstream region (500 bp respectively) of the gene *relS* from *C. glutamicum* CR099	This study
pRedET	Red/ET expression plasmid	Gene Bridges
708-FLPe	FLPe expression plasmid	Gene Bridges
pTXB1	*E. coli* expression vector, carrying the self-cleavable *Mxe* intein/chitin binding domain	New England Biolabs
pTXB1::relP^*^_Cg_	pTXB1 carrying the gene relP^*^ (*cg1330*) from *C. glutamicum* CR099	This study
pTXB1::*relS*_Cg_	pTXB1 carrying the gene *relS* (*cg2324*) from *C. glutamicum* CR099	This study
pTXB1::*rel*_Cg_	pTXB1 carrying the gene *rel* (*cg1861*) from *C. glutamicum* CR099	This study

*Corynebacterium glutamicum* cells were routinely grown in CASO broth at 30°C. The defined CGXII mineral salt medium (Keilhauer et al., 1993) with a glucose concentration of 10 g L^−1^ was used in addition to the complex CASO medium for growth experiments, which were carried out in a 2 inch deviation Innova 44R shaker (New Brunswick), using 250 mL shaking flasks and a shaking frequency of 300 rpm. All cultures were inoculated from precultures in complex CASO-broth. An initial OD_600_ of 0.2 was used for cultivation in CASO-broth. For growth analysis in CGXII minimal medium, precultures were washed in main culture CGXII medium and then diluted to an initial OD_600_ of 0.5. Unless stated otherwise, *E. coli* cells were cultured in LB broth at 37°C. Kanamycin (50 μg mL^−1^) and ampicillin (100 μg mL^−1^) were used as selection markers.

### Construction of strains

All primers used in this study were designed with Clone Manager Professional 9 (Scientific & Educational Software) and are listed in Table S1 with their respective sequences and purposes. For the construction of pK18*mobsacB*-based deletion constructs, approximately 500 bp long regions up- and downstream of the target gene were amplified by PCR and cloned into the vector backbone by Gibson Isothermal Assembly (Gibson et al., 2009). All constructs were introduced into *C. glutamicum* CR099 cells by means of electroporation. Preparation of electrocompetent *C. glutamicum* cells and electrotransformation was performed as described by Tauch et al. for *Corynebacterium diphtheriae* (Tauch et al., 2002), screening for the first and second recombination events were performed as described previously (Schäfer et al., 1994). Both kanamycin-sensitive and sucrose-resistant clones were examined by colony PCR analysis, using primers that bind 250 bases up- and downstream of the flanks used. The construction of *C. glutamicum* CR099 double and triple deletion mutants was based on the previously generated single or double deletion mutants using the identical procedure.

The construction of *E. coli* deletion mutants was performed using the Quick & Easy *E. coli* gene deletion kit (Gene Bridges). The procedure was carried out according to the manufacturer's instructions using the FRT-PGK-gb2-neo-FRT template DNA. By choosing appropriate short homologous sequences, which were attached to the functional cassette by PCR, the genes *relA* and *spoT* could be removed consecutively in the form of start-stop deletions.

### Complementation test for (p)ppGpp synthetase activity in *E. coli*

To generate expression constructs for the different stringent response-associated genes from *C. glutamicum* CR099 and *C. glutamicum* AS1.542, the corresponding genes *relS*_Cg_, relP^*^_Cg_, *rel*_Cg_, and *relP*_Cg_ were cloned into the vector pBAD24 by Gibson Isothermal Assembly (Gibson et al., 2009). The resulting plasmids pBAD::*relS*_Cg_, pBAD::relP^*^_Cg_, pBAD::*rel*_Cg_ and pBAD::*relP*_Cg_ were subsequently transformed into the *E. coli* Δ*relA*Δ*spoT* strain.

Since the addition of low arabinose concentrations had no effect on the growth of the investigated strains in complex medium, all cultures were spiked with a low arabinose concentration of 0.001% (m/v) during cultivation in LB medium to ensure basal expression of the target genes. All strains were inoculated with an OD_600_ of 0.05 and incubated for 3 h at 37°C. After cultivation, the cells were washed and diluted in PBS buffer (pH 7.4) to identical cell titers. To investigate the growth characteristics of the different strains, 5 μL aliquots of various dilution stages were spotted onto minimal medium plates (Kasai, 2002), containing 4 g L^−1^ glycerol and different arabinose concentrations. The plates were incubated at 37°C for 48 h.

### Protein purification

The IMPACT kit (New England Biolabs) was used for the tag-free purification of the proteins RelS_Cg_, RelP^*^_Cg_, and Rel_Cg_. The corresponding genes *relS*_Cg_, relP^*^_Cg_, and *rel*_Cg_ were introduced into the vector pTXB1 by Gibson Isothermal Assembly and the obtained plasmids were transformed into the expression strain *E. coli* ER2566. The cultivation of the resulting strains was carried out in shaking flasks, using LB broth and an initial OD_600_ of 0.1. After a 2-h cultivation period at 37°C, protein expression was induced by the addition of IPTG (400 μM final concentration). To avoid inclusion bodies, the cultivation temperature was reduced to 19°C 30 min after induction. Following a cultivation period of 16 h, the cells were centrifuged and resuspended in lysis buffer [25 mM Tris-HCl (pH 8.5), 500 mM NaCl, 1 mM EDTA, 0.15% (w/v) Triton X-100, 20 μM PMSF, and 1 mM TCEP]. A French press was used for cell disruption in two runs at about 1,200 psi. The chitin resin based column purification of the heterologously expressed proteins was carried out according to the stated manufacturer data, using a column buffer composed of 25 mM Tris-HCl (pH 8.5), 500 mM NaCl, and 1 mM EDTA. To wash the column, a high salt column wash buffer with a NaCl concentration of 1,500 mM was used. The eluate was concentrated using a 10 kDa cut-off centrifugal filter unit and then dialyzed twice against a glycerin-containing storage buffer [300 mM NaCl, 50 mM Tris-HCl, pH 8.5, 50% (m/v) glycerol] in a 10 kDA cut-off dialysis cassette. Roti-Nanoquant (Roth) was used to determine the protein concentration and storage was carried out at −20°C.

### (p)ppGpp synthetase assays

In order to analyze the biochemical properties of the purified proteins, they were incubated with various substrates under variation of relevant parameters. All assay reactions were carried out in a total volume of 50 μl and the enzyme activity was inactivated after the reaction by the addition of 10 μL of glacial acetic acid. Enzyme free controls and nucleotide standards measured for concentration determination were treated analogously.

The reaction buffer for determining the activity kinetics with respect to various substrate concentrations contained 50 mM HEPES-Na (pH 7.0), 200 mM NaCl, and 5 mM MgCl_2_. 25 mM ATP and varying guanosine derivate concentrations were applied to determine substrate concentration dependency. For the analysis of the kinetics with respect to the substrate ATP, (p)ppGpp synthetase assays with a GTP concentration of 25 mM and various ATP concentrations were performed. 250 nM enzyme was used and the reaction was carried out at 30°C for 60 min.

The general activity analysis of the three purified enzymes as well as the characterization of RelS_*Cg*_ and Rel_*Cg*_ with respect to relevant activity parameters was based on pH optimum values of bifunctional RSH enzymes at pH 8 (Sajish et al., 2007), a physiological Mg^2+^ concentration of 5 mM (Nierhaus, 2014) and the standard *C. glutamicum* growth temperature of 30°C. The reaction mixture contained 50 mM Tris-HCl (pH 8.0), 5 mM MgCl_2_, 4 mM ATP, and 4 mM GTP/GDP/GMP as well as 500 ng of the respective enzyme and was incubated for 4 h at 30°C. For the further analysis of RelS_Cg_ and Rel_Cg_ the individual influence factors temperature, MgCl_2_ concentration and pH were varied using GTP as guanosine substrate. In order to achieve a broad spectrum of different pH values, citrate, phosphate, Tris and ammonia buffer systems with a concentration of 50 mM each were used.

HPLC analysis of all assay reactions was performed using a LaChrome ULTRA system (HITACHI) and a SeQuant ZIC-pHILIC column (Merck Millipore). A MicrOTOFQ (Bruker Daltonics) was used for the mass spectroscopic identification of the different nucleotide species. The individual components were separated by means of an isocratic elution profile with 38% of 10 mM ammonium bicarbonate buffer (pH 9.3) and 62% of acetonitrile over a period of 30 min at a flow rate of 0.2 mL min^−1^. The injection volume was 1 μL and the detection was carried out at a wavelength of 252 nm. For the determination of pGpp, ppGpp and pppGpp concentrations, the equimolar produced by-product AMP was quantified since more exact and reproducible UV peaks were obtained for this component and no suitable standards were available for the three alarmone species. In order to analyze the activity spectra of the investigated enzymes, the peaks of the UV chromatograms were identified using the MS data. The respective peak areas were first corrected by the results of an enzyme-free negative control and then normalized to the protein amounts and reaction times.

Data analysis was performed using the program OriginPro 2017 (OriginLab). For the determination of the enzyme kinetic parameters v_max_ and K_m_, polynomial fits for the relationships between specific activity and the substrate concentrations were carried out due to product excess inhibition.

## Results

### The *C. glutamicum* genome encodes two putative small alarmone synthetases (SAS)

In addition to the already known *rel*_Cg_ gene (Wehmeier et al., 1998), three further putative stringent response-associated open reading frames were identified in the genome of *C. glutamicum* by Atkinson et al. in the course of a high-throughput sensitive sequence analysis of the RSH superfamily (Atkinson et al., 2011). Two of the putative ORFs (*cg1330* and *cg2324*) were assigned to different SAS classes and one (*cg1485*) to the SAHs. In this study, we focused on the investigation of the two putative monofunctional pyrophosphokinases with the aim of identifying novel (p)ppGpp sources in *C. glutamicum*.

**Figure 1:**
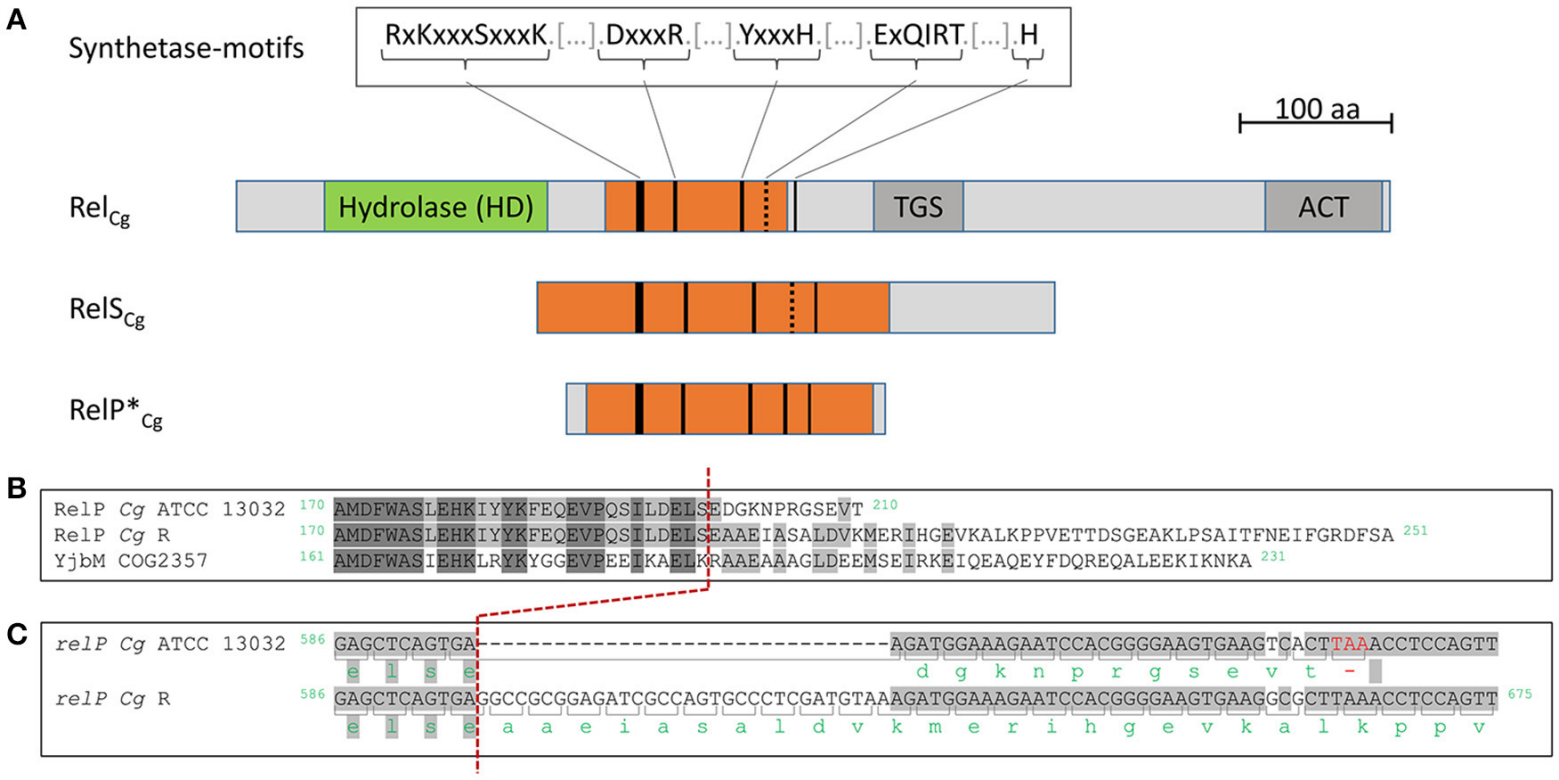
*C. glutamicum* enzymes involved in (p)ppGpp synthesis. **(A)** True-to-scale representation of domain architecture and conserved sequence motives of stringent control-associated enzymes of *C. glutamicum* ATCC 13032. Hydrolase domain (pfam01966) and ppGpp synthetase catalytic domain YjbM (COG2357) are indicated in green and orange, respectively. Matches with conserved synthetase motifs are represented by black lines (Bag et al., 2014; Steinchen and Bange, 2016). Minor deviations from the conserved sequence motifs are shown by dashed black lines. **(B)** Sequence alignment of the C-termini of RelP variants from *C. glutamicum* ATCC 13032 (*Cg* ATCC13032) and *C. glutamicum* R (*Cg* R) with the synthetase catalytic domain consensus sequence YjbM (COG2357). Identical residues among all three sequences are indicated by dark shading and matches between two sequences by light shading, respectively. Amino acid positions are shown at both sequence ends. **(C)** Alignment of the nucleotide sequences of the *relP* variants from *C. glutamicum* ATCC 13032 and *C. glutamicum* R. Codons and the corresponding amino acid residues are indicated below the nucleotide sequences with stop codons highlighted in red. Nucleotide positions are shown at both sequence ends. Since the *relP* sequence of *C. glutamicum* ATCC 13032 ends before the end of the alignment, no positional information can be given. Matches between the nucleotide and protein sequences are represented by gray shadings. The relationship between the nucleotide sequences and the corresponding amino acid sequences is illustrated by a dashed red line.

Based on a phylogenetic analysis of the RSH superfamily, carried out by Atkinson et al., the gene *cg1330* of *C. glutamicum* ATCC 13032 was classified as a member of the RelQ subgroup (Atkinson et al., 2011). However, an in-depth investigation of the corresponding protein sequence indicated that Cg1330 has similarities with SAS enzymes from *S. mutans* and *B. subtilis*, which are referred to as RelP/SAS2. Since the corresponding proteins were also classified as representatives of the SAS RelQ subfamily by Atkinson et al. (2011), a mix-up of the original nomenclature seems to exist at this point. The same holds true the other way around, proteins originally labeled as RelQ were classified as representatives of the SAS subgroup RelP, so that the names RelP and RelQ were evidently interchanged in the course of the classification of the RSH superfamily by Atkinson et al. (2011). In order to avoid inconsistency with the already performed analyzes of the SAS proteins, the gene product of *cg1330* is hereinafter referred to as RelP_Cg_. A BLASTP analysis of the corresponding protein sequence illustrated, that this subgroup occurs almost exclusively in Firmicutes and that *C. glutamicum* is the sole member of the Corynebacteriaceae possessing a RelP variant (Camacho et al., 2009). In analogy to the bifunctional Rel_Cg_ enzyme of *C. glutamicum*, RelP_Cg_ exhibits high similarities to five synthetase sequence motifs, which are strongly conserved for RSH proteins in other Actinobacteria, such as Mycobacteria, in Firmicutes, in Proteobacteria, and in plants (Bag et al., 2014; Steinchen and Bange, 2016; Figure 1A).

In the course of our protein sequence analysis, it was found that the known *C. glutamicum* genomes show differences with respect to the *cg1330* (*relP*) gene. In comparison to other strains like *C. glutamicum* R (Yukawa et al., 2007), *C. glutamicum* ATCC 13032 features a RelP version that is shortened by 41 aa at its C-terminus and has an alternative 11 aa C-terminal end sequence (Figure 1B). An alignment with the ppGpp synthetase catalytic domain YjbM consensus sequence illustrates the special position of the ATCC 13032 RelP variant. The shorter version of RelP_Cg_ that is present exclusively in ATCC 13032 and its derivatives shows all SAS synthetase protein motifs but lacks some highly conserved amino acids located in its C-terminal region. These highly conserved amino acid residues are present in the genomes of other *C. glutamicum* isolates (Figure 1B). A multiple alignment of the amino acid sequence of potential homologs of RelP_Cg_ selected based on amino acid sequence similarity emphasizes this feature since the RelP protein from *C. glutamicum* ATCC 13032 has the shortest of all found RelP variants (Figure S1). The difference was further investigated by an alignment of the corresponding nucleotide sequences. The analysis identified a 32 bp deletion for the ORF *cg1330* from *C. glutamicum* ATCC 13032 compared to the otherwise largely identical sequence of the strain *C. glutamicum* R (Figure 1C). This deletion leads to a frameshift, starting from amino acid position 199, and the formation of an alternative stop codon and explains the differences between the protein sequences within the different *C. glutamicum* strains. Based on this result, the *cg1330* gene of the strain *C. glutamicum* ATCC 13032 is henceforth referred to as relP^*^_Cg_, while the other *C. glutamicum* variant is termed *relP*_Cg_.

The second putative SAS gene, *cg2324*, was assigned to the actRel subgroup of the small SASs (Atkinson et al., 2011). Since there is no appropriate gene name for members of this group, we here propose the term *relS*, with the *C. glutamicum* actRel protein being encoded by *relS*_Cg_. The amino acid sequence of this protein also shows a high similarity to the ppGpp synthetase catalytic domain YjbM (COG2357) over a range of 231 aa, and contains all conserved synthetase motifs (Steinchen and Bange, 2016; Figure 1A). Interestingly, both RelS_Cg_, and Rel_Cg_ show slight deviations from the highly conserved Syn4 amino acid sequence motive ExQIRT (Bag et al., 2014; Steinchen and Bange, 2016). To date, only one representative of the actRel subgroup has been studied in more detail, the enzyme MS_RHII-RSD from *Mycobacterium smegmatis* (Murdeshwar and Chatterji, 2012). However, due to an additional RNase HII domain, this 64.5 kDa enzyme is significantly larger than RelS_Cg_, which has only a molecular weight of 38.9 kDa. The additional RNase domain was also found in further representatives of the subgroup (Atkinson et al., 2011). To our knowledge, RelS_Cg_ therefore is the first analyzed representative of the actRel subgroup without an RNase domain.

### Phylogeny of RelS homologs

In order to analyze the phylogenetic affiliation of the protein RelS_Cg_ more precisely, a phylogenetic tree of RelS homologs was created (Figure S2). In addition to proteins with sequence similarity to RelS_Cg_, proteins allocated to the actRel subgroup by Atkinson et al. were taken into account during the analysis (Atkinson et al., 2011). In general it was found that a RelS homolog is only present in species belonging to the phylum Actinobacteria. In contrast to RelP, the actRel subgroup is abundant in the family Corynebacteriaceae and the corresponding proteins can be distinguished phylogenetically from further homologous representatives. As already demonstrated by Murdeshwar and Chatterji, some species of the closely related genus *Mycobacterium* and the more remote genus *Marmoricola* possess RelS variants for which a second domain, namely the RNase H-associated protein domain COG4328, was identified. It is interesting to note that all mycobacteria which contain this fusion protein (N-terminal RNase H domain and a C-terminal (p)ppGpp synthetase domain) represent exclusively environmental species while it appears to be absent in the representatives of the human pathogenic *Mycobacterium tuberculosis* (Mtb)-complex. In addition to the two large groups already described, many other actinobacterial genomes also contain a *relS* homolog. However, the performed phylogenetic analysis does probably not represent the complete picture as all candidates were identified only by similarity to RelS_Cg_. This is particularly clear from the fact that some representatives of the actRel subgroup according to Atkinson et al. are located far outside the phylogenetic RelS tree. For that reason, this approach should be extended and ideally tested experimentally.

### The growth deficit of a *C. glutamicum* Δ*rel* strain is compensated by the additional deletion of *relS*_Cg_; the deletion of relP^*^_Cg_ has no effect

Since the deletion of the *rel* gene (*cg1861*) was known to have a negative effect on the growth of *C. glutamicum* in a chemically defined minimal medium (Tauch et al., 2001), deletion mutants missing the putative monofunctional (p)ppGpp synthetases RelP^*^_Cg_ or RelS_Cg_ were analyzed for growth defects. The corresponding genes as well as *rel*_Cg_ were removed individually from the genome of the strain *C. glutamicum* CR099 (Unthan et al., 2015) by homologous recombination, using the vector pK18*mobsacB* (Schäfer et al., 1994). The strain CR099 is a direct descendant of ATCC 13032 but devoid of three prophages (ΔCGP1/2/3) and all members of two IS-element families (ISCg1 and ISCg2). In order to prevent enrichment of suppressor mutations in precultures of the growth experiment, as observed for *E. coli* ppGpp^0^ strains if cultivated in minimal medium (Murphy and Cashel, 2003), all precultures were grown in complex CASO medium. To analyze the growth characteristics in minimal medium, the precultures were then washed in the main culture CGXII medium and adjusted to the desired inoculum density.

**Figure 2:**
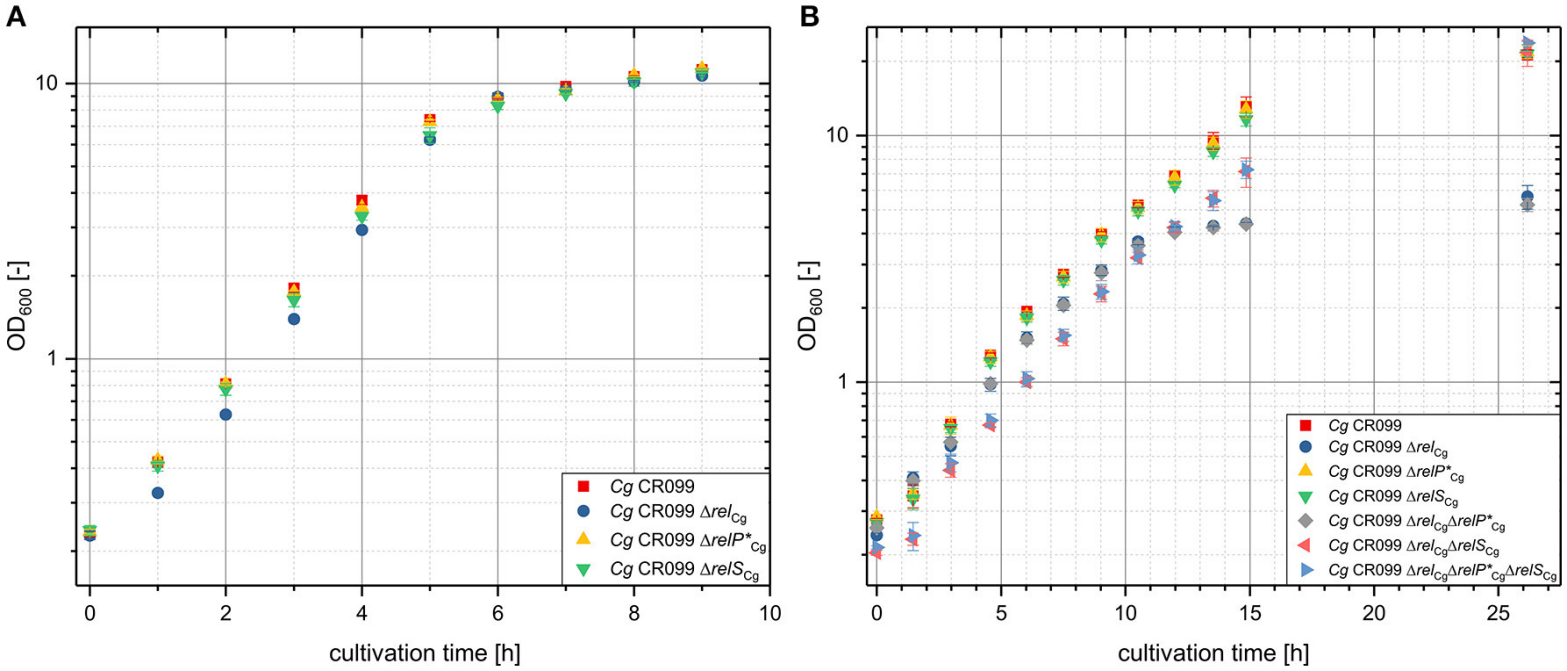
Effect of the deletion of genes with a putative (p)ppGpp synthetase motive on the growth of *C. glutamicum* CR099 in complex CASO-broth **(A)** and minimal CGXII medium **(B)**. To preclude possible enrichment of suppressor mutants all cultures were inoculated from precultures in complex CASO-broth. An initial OD_600_ of 0.2 was used for the cultivation in CASO-broth. For the growth analysis in CGXII minimal medium, the precultures were washed in the main culture medium and then diluted to an OD_600_ of 0.5. Mean values and standard deviations shown were calculated from three biological replicates.

No significant growth differences were observed for the three analyzed single deletion mutants in complex CASO medium (Figure 2A). Although the strain CR099Δ*rel* showed slightly delayed growth, the growth rate during exponential growth phase was equivalent to that of the parent strain CR099 and to the deletion mutants CR099 ΔrelP^*^_Cg_ and CR099 Δ*relS*_Cg_.

Since the deletions of relP^*^_Cg_ and *relS*_Cg_ also did not lead to a change in growth behavior during the cultivation in minimal medium (Figure 2B), both putative monofunctional SAS enzymes seem to be dispensable individually under standard growth conditions.

While in a previous study (Tauch et al., 2001), a partial *rel*-deletion mutant based on the strain *C. glutamicum* RES167 showed a strong growth defect and requirements for L-histidine and L-serine, the complete deletion of *rel* in *C. glutamicum* CR099 caused a different growth phenotype in CGXII minimal medium (Figure 2B). In the first phase of the cultivation the strain showed almost no growth difference to the parental strain. In the course of the growth experiment, however, a continuous reduction of the growth rate occurred in contrast to the parental strain. After a cultivation period of about 12 h, strain CR099 Δ*rel* almost reached a stationary state with an OD_600_ of 4. Although the OD_600_ increased only slightly to a value of 5 after a cultivation period of 26 h, the strain reached the same end OD_600_ as the parental strain after 48 h.

In order to analyze the *in vivo* role of RelS_Cg_ and RelP^*^_Cg_ or possible interrelations between Rel_Cg_ and the putative SAS enzymes, the growth experiment in minimal medium was extended by the strains CR099 Δ*rel*ΔrelP^*^ and CR099Δ*rel*Δ*relS* as well as the triple deletion mutant CR099 Δ*rel*ΔrelP^*^Δ*relS* (Figure 2B). For multiple deletions involving the gene relP^*^_Cg_, no change in the growth behavior was observed. The strain CR099 Δ*rel*ΔrelP^*^ behaved identically to the strain CR099 Δ*rel* and the triple deletion mutant grew with the same characteristic as the strain CR099 Δ*rel*Δ*relS*. Thus, a deletion of gene relP^*^_Cg_ does not seem to have any physiological effects under the analyzed growth conditions.

In contrast to this, the simultaneous absence of *rel*_Cg_ and *relS*_Cg_ in the strains CR099 Δ*rel*Δ*relS* and CR099 Δ*rel*ΔrelP^*^Δ*relS* caused a clear growth effect (Figure 2B). In the first hours of cultivation, both strains showed a short lag phase in contrast to all other strains examined and started growing after 1.5 h with a growth rate corresponding to that of the wild type. In the further course, both mutants showed uniform growth and did not show the growth rate reduction as observed for strain CR099 Δ*rel*. The comparison of the corresponding growth characteristics clearly shows an *in vivo* role of RelS_Cg_.

Since the growth rate reduction of the strain CR099 Δ*rel* can be compensated by an additional deletion of *relS*_Cg_, the expression of RelS_Cg_ appears to be a problem for the cells in the strain without the bifunctional enzyme Rel_Cg_.

### Expression of RelS_Cg_ but not RelP^*^_Cg_ complements the multiple amino acid auxotrophy of *E. coli* Δ*relA*Δ*spoT*

In order to analyze the *in vivo* functionality of the identified putative SAS enzymes RelP^*^_Cg_ and RelS_Cg_, a complementation experiment of an *E. coli* Δ*relA*Δ*spoT* strain derived from the K12 wild-type MG1655 strain was attempted. The *relA spoT* double deletion mutant is a (p)ppGpp^0^ strain, which shows multiple amino acid auxotrophies and therefore can no longer grow in minimal medium (Xiao et al., 1991). In order to realize different expression levels of the proteins of interest in this strain, the corresponding genes were cloned into the arabinose-inducible vector pBAD24. This allows a precise control of the strength of expression by the variation of inducer concentration as well as the used carbon source (Guzman et al., 1995). In addition to the two genes *relS*_Cg_ and relP^*^_Cg_, the gene *rel*_*Cg*_, coding for the *C. glutamicum* ATCC 13032 RSH enzyme and the non-truncated *relP*_Cg_ variant from the strain *C. glutamicum* AS1.542 were cloned into the vector pBAD24.

**Figure 3:**
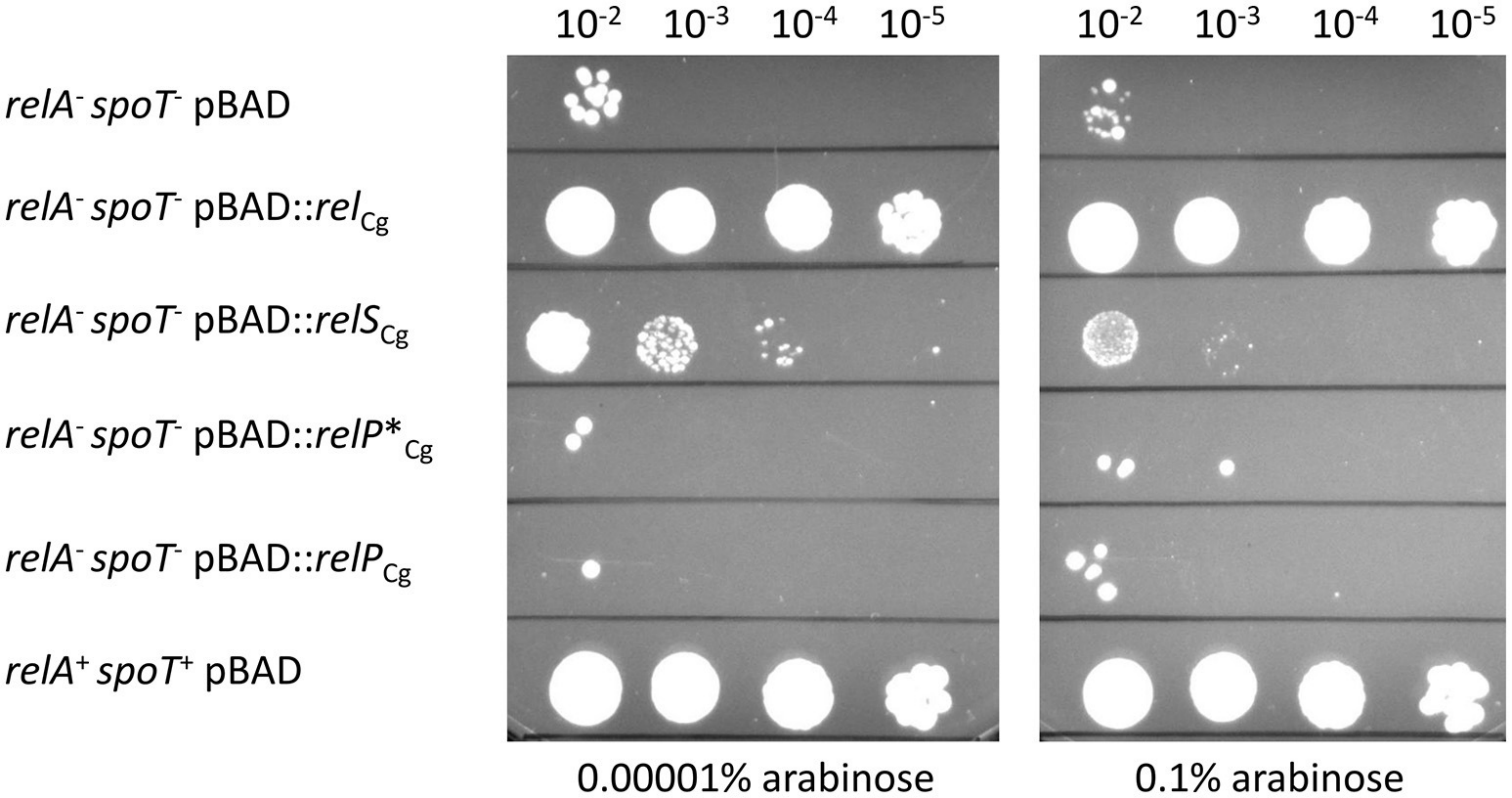
Growth complementation of *E. coli* Δ*relA*Δ*spoT* on minimal medium plates by heterologous expression of *C. glutamicum* genes *rel*_Cg_, *relS*_Cg_, relP^*^_Cg_ or *relP*_Cg_. The parental strain *E. coli* MG1655 and *E. coli* Δ*relA*Δ*spoT* each containing the empty vector pBAD24 were used as a positive and negative control, respectively. All strains were cultivated using initial OD_600_ values of 0.05 in LB-Medium containing 0.001% arabinose for 3 h. The cells were washed and serially diluted in PBS-buffer. 5 μL aliquots of every strain and dilution stage were spotted on minimal medium plates with 0.4% glycerol as carbon source and different arabinose concentrations. Incubation was carried out at 37°C for 48 h.

As expected, the *E. coli* Δ*relA*Δ*spoT* strain containing the empty plasmid control pBAD showed only single colonies in the lowest dilution stage of 10^−2^, whereas the parental *E. coli* strain MG1655 with the same plasmid formed numerous colonies even in the highest dilution stage of 10^−5^ (Figure 3). The non-auxotrophic colonies of the double deletion strain are all likely to be (p)ppGpp^0^ suppressor phenotypes, also called *E. coli* M+ mutants, which are pseudo-revertants generated by spontaneous mutations (Murphy and Cashel, 2003). A corresponding picture of individual revertants was also shown for both RelP variants RelP^*^_Cg_ and RelP_Cg_. Therefore, no complementation effect could be found for these proteins. In contrast, the expression of the gene *relS*_Cg_ resulted in a complementation of the auxotrophic test strain even at very low arabinose concentrations of 0.00001%. Since the auxotrophy of the complemented strain is due to the absence of a basal (p)ppGpp level, RelS_Cg_ thus most probably provides a (p)ppGpp synthetase activity. While significant growth was still evident at this low inducer concentration up to the dilution factor 10^−4^, an increased inducer concentration of 0.1% arabinose led to a clearly weaker complementation with significantly smaller colonies up to dilution stage 10^−3^. The expression of the RSH protein-encoding gene *rel*_Cg_, on the other hand, led to a complete and inducer concentration-independent complementation of the amino acid auxotrophy, since the corresponding strain showed no difference to the non-auxotrophic parental strain *E. coli* MG1655 for all tested inducer concentrations.

### *In vitro* analysis of (p)ppGpp synthetase activity of RelP^*^_Cg_, RelS_Cg_ and Rel_Cg_

In order to investigate the catalytic activities of the putative monofunctional small SASs, RelS_Cg_, and RelP^*^_Cg_, both enzymes as well as the RSH-protein Rel_Cg_ were analyzed biochemically by means of *in vitro* synthetase assays. The NEB IMPACT™ system was used for the tag-free preparation of all enzymes, which were successfully checked for purity and identity by SDS-PAGE and MALDI-TOF analysis (data not shown). For a precise evaluation of (p)ppGpp synthetase assays, which were based on established reaction conditions, a LC-MS approach was developed. Compared with the routinely used TLC-based analysis of radioactively labeled nucleotides, this method allows a clear identification and more accurate quantification of the reaction products.

**Figure 4:**
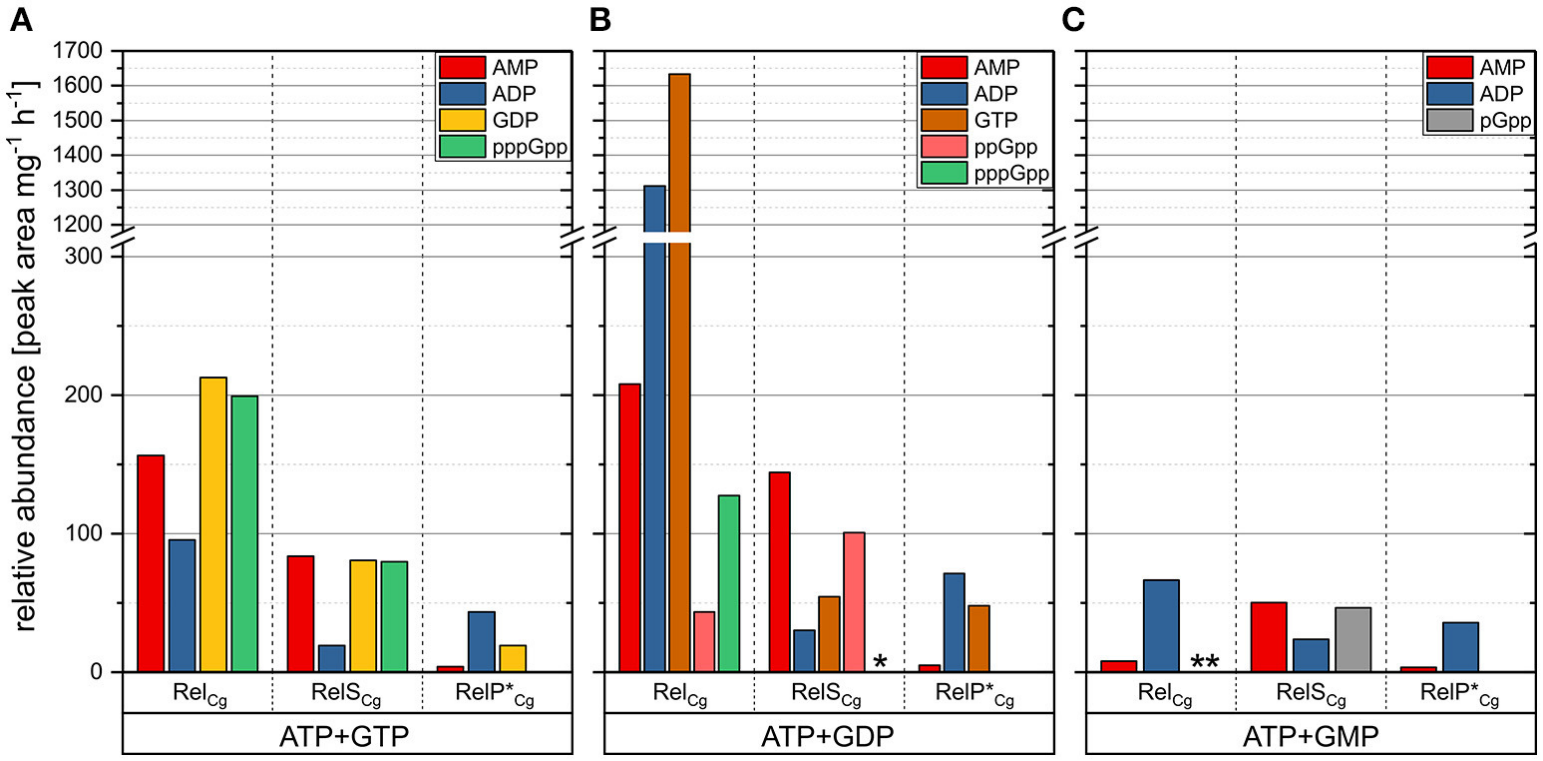
*In vitro* activity characterization of the putative (p)ppGpp synthetases RelP^*^_Cg_ and RelS_*Cg*_, as well as the enzyme Rel_*Cg*_. Graphical representation of the HPLC analysis of 50 μL assay reactions containing 500 ng of the corresponding enzyme and ATP+GTP **(A)**, ATP+GDP **(B)** and ATP+GMP **(C)** as substrate combinations with a concentration of 4 mM each. HPLC separation was performed using a SeQuant ZIC-pHILIC column and isocratic elution with 38% of 10 mM ammonium bicarbonate buffer (pH 9.3) and 62% acetonitrile. The reaction products were identified by their specific masses and retention times. Their relative abundance is given as the peak area of the UV signal (252 nm) per mg of the respective enzyme per hour. The results shown were corrected by the values determined for enzyme-free controls. For some measurements pppGpp (^*^) or pGpp (^**^) were detected by their characteristic MS signals, but the UV detection limits were not reached.

In the reaction mixture containing RelP^*^_Cg_ as well as ATP and GTP as substrates, significant amounts of ADP and GDP were detected (Figure 4A). This result implies a phosphohydrolase activity of RelP^*^_Cg_. Furthermore, RelP^*^_Cg_ possesses a kinase activity, since ADP and GTP were produced in the assay including ATP and GDP (Figure 4B). In addition, very small amounts of AMP were detected in all assay reactions containing RelP^*^_Cg_ (Figure 4). Despite the very sensitive MS signal, however, no evidence for the production of an alarmone species was found. For this reason, the reaction product AMP apparently is not due to a pyrophosphokinase reaction but results from enzymatic degradation of ADP. To exclude inappropriate reaction conditions as reason for the missing pyrophosphokinase activity, different buffer systems and salt concentration were tested in compliance with positive characterizations of RelP homologs and further SAS enzymes from other organisms (data not shown). However, no alarmones were synthesized in any of the different conditions, indicating that RelP^*^_Cg_ lacks the suggested activity. The result of the *in vitro* analysis thus complies with the previously performed complementation experiments in which no *in vivo* (p)ppGpp synthetase activity could be detected for the corresponding enzyme RelP^*^_Cg_, as well as the non-shortened version RelP_Cg_.

For the second putative SAS RelS_Cg_, the proposed pyrophosphokinase activity was clearly identified. In the presence of this enzyme, a high amount of pppGpp was detected in the reaction mixture containing ATP and GTP as substrates (Figure 4A). In addition to AMP, which is also produced during the pyrophosphokinase reaction, ADP and GDP were detected. This suggests that RelS_Cg_ has a phosphohydrolase activity similar to RelP^*^_Cg_. Using ATP and GDP as substrates, ppGpp was synthesized in the reaction mixture containing RelS_Cg_(Figure 4B). Interestingly, for the synthetase assay reaction with ATP and GMP as substrates, a mass-to-charge ratio of 521.99 was found as a product which exactly complies with the corresponding value of GTP. However, since the retention time slightly differed from the one found for GTP and the reaction mechanism of the enzyme seems to be selective for the pyrophosphorylation in the 3′-position, the detected product is thought to be GMP 3′-diphosphate (pGpp) (Figure 4C).

According to expectations, the pyrophosphorylation of GDP and GTP to ppGpp and pppGpp was detected for the bifunctional RSH enzyme Rel_Cg_ (Figures 4A,B). Interestingly, very high ADP and GTP concentrations were observed using ATP and GDP as substrates (Figure 4B). The enzyme Rel_Cg_ thus apparently has a kinase activity since this product spectrum can be explained by the transfer of the terminal phosphate from ATP to GDP. Furthermore, this assay reaction contained a mixture of ppGpp and pppGpp. With the help of the sensitive MS signal and an exact evaluation of the retention time, pGpp could be detected for the substrate combination ATP/GMP (Figure 4C). However, due to the very low amount of pGpp, which was below the UV detection limit, the corresponding enzyme activity of Rel_Cg_ is very low.

Since a (p)ppGpp synthetase activity could not be detected for RelP variants from *C. glutamicum*, neither in the *in vivo* complementation experiments nor in the *in vitro* assays, we focused on the more detailed analysis of the enzyme RelS_Cg_.

### RelS_Cg_ shows unusual enzyme kinetics with high K_m_ values for GTP, GDP, and GMP

In order to characterize RelS_Cg_ in more detail, a comparison of the enzyme activity for various substrates was done. It was recently shown that the reaction products pGpp, ppGpp and pppGpp exhibit slightly different biological functionalities (Mechold et al., 2013). Different enzyme activities with respect to the possible substrates could therefore have a great influence on the biological significance of the enzyme in the context of the stringent response.

To investigate the enzyme kinetics, the specific pyrophosphokinase activity of RelS_Cg_ was determined for different substrate concentrations. Physiological reaction conditions with a pH of 7.0 as well as a Mg^2+^ concentration of 5 mM, which was found for *E. coli* (Nierhaus, 2014), were applied. To be able to compare the generated data with known kinetic parameters of other SAS enzymes, further assay components as well as the buffer system used were adapted to the corresponding studies (Murdeshwar and Chatterji, 2012; Steinchen et al., 2015). Using standard substrate concentrations of up to 5 mM, no substrate saturation of the specific pyrophosphokinase activity was achieved for all substrates. Nevertheless, in order to determine parameters of the RelS kinetics, higher GMP/GDP/GTP concentrations were used and the ATP concentration was adjusted to 25 mM to exclude a limitation of the second substrate. To investigate the influence of ATP, kinetics for the conversion of ATP and GTP to pppGpp were recorded with different ATP concentrations. Since the half-maximal specific activity has already been reached for approximately 0.8 mM ATP, this substrate seems not to be rate-determining in comparison with guanosine substrates (Figure S3).

**Figure 5:**
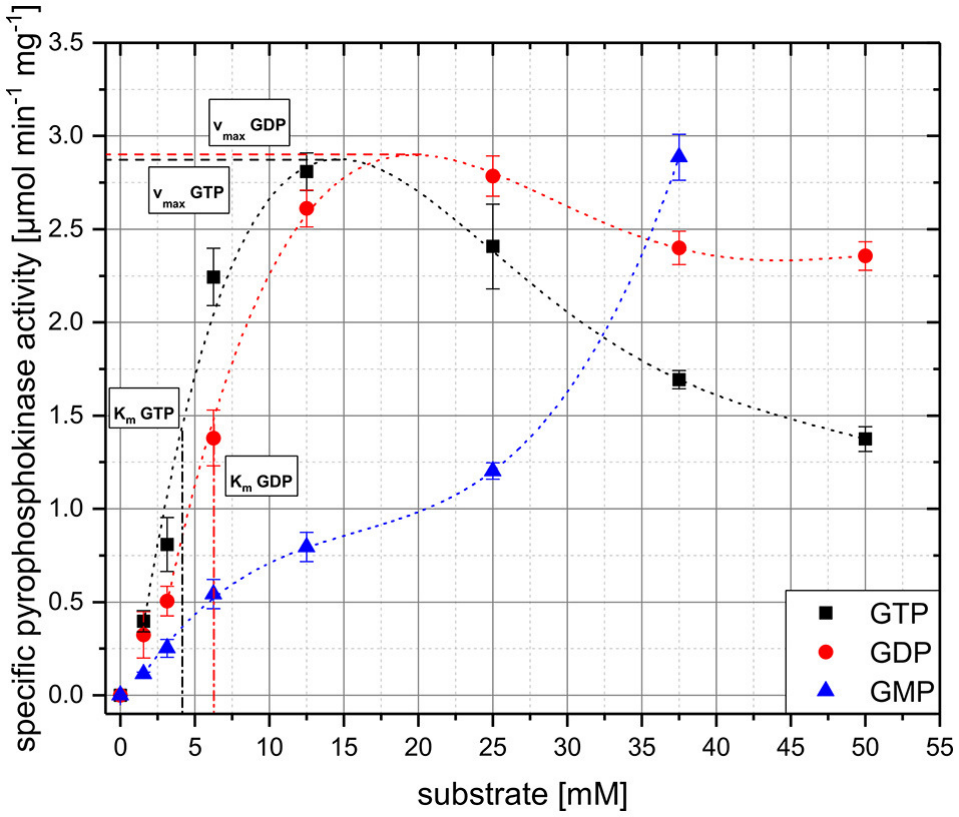
Kinetics of RelS_Cg_ with respect to the concentrations of GMP, GDP, and GTP as substrates. Specific activity was determined for different substrate concentrations by *in vitro* analysis and given in μmol per minute per milligram of RelS_Cg_. In order to illustrate the data and the graphical determination of the kinetic parameters K_m_ and v_max_, a polynomial fit was performed for all substrates and displayed as a dashed line. K_m_ and v_max_ values for GDP and GTP are represented as vertical and horizontal dashed lines, respectively. For GMP the parameters could not be determined due to the untypical activity profile. Mean values and standard deviations shown were calculated from three replicates.

Similar kinetics with nearly identical maximum specific pyrophosphokinase activities (v_max_) of approximately 2.8 μmol min^−1^ mg^−1^ were determined for either GTP or GDP (Figure 5). However, using GDP v_max_ is reached at significantly higher concentrations of 20 mM, compared to GTP for which a value of 14 mM was determined. Furthermore, RelS achieves its half-maximal pyrophosphokinase activity for the conversion of GTP to pppGpp at a substrate concentration of 4.1 mM, whereas a higher K_m_ value of 6.1 mM was determined for the substrate GDP. At very high substrate concentrations, a reduced pyrophosphokinase activity with respect to GDP and GTP was observed.

RelS shows a very untypical kinetics for GMP. At low substrate concentrations of up to about 7 mM, a nearly linear increase in activity takes place, whereby the v_max_ values are significantly lower than for the other substrates. At higher substrate concentrations of 10–25 mM, a saturation effect occurs analogous to the kinetics for GDP and GTP. In the case of even higher substrate concentrations, however, the specific pyrophosphokinase activity increases significantly again to values around 2.8 μmol min^−1^ mg^−1^. Using a GMP concentration of 50 mM the addition of acetic acid to stop the reaction resulted in an increased viscosity, so that the sample could no longer be properly injected. Since the gelatinous consistency also occurred in the negative control without ATP, as well as without addition of enzyme it seems to be a non-pGpp or RelS associated effect.

### RelS_Cg_ is highly Mg^2+^ dependent and most active at neutral pH conditions and at low temperatures

**Figure 6:**
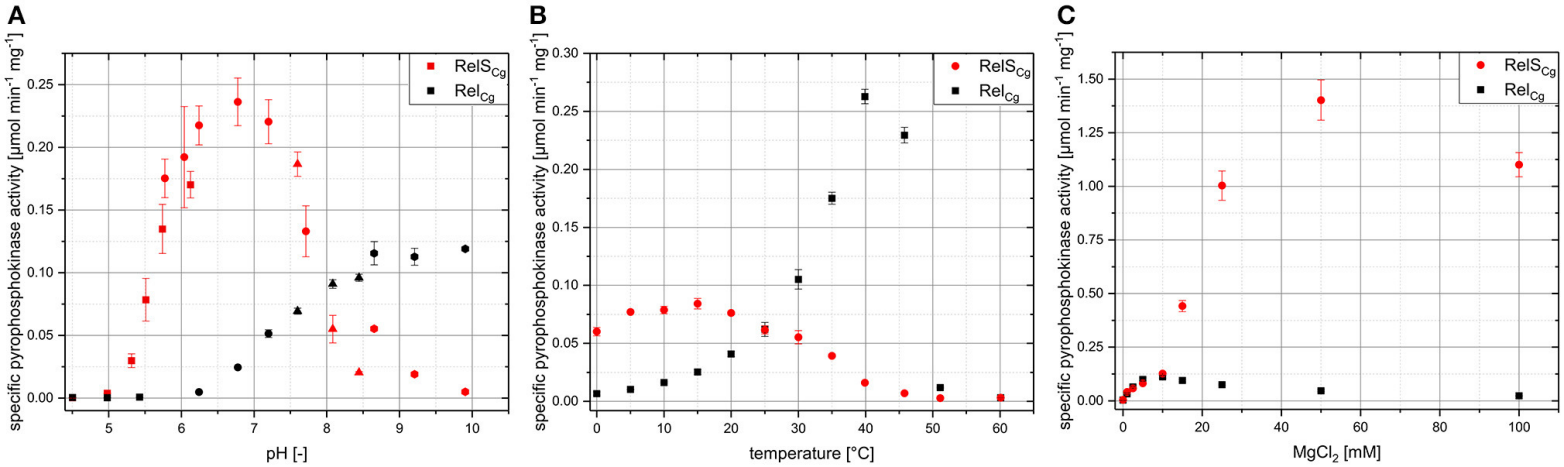
Analysis of relevant reaction parameters on the activity of RelS_Cg_ and Rel_Cg_. Specific pyrophosphokinase activities were determined by *in vitro* analysis under variation of pH-value **(A)**, temperature **(B)**, and MgCl_2_ concentration **(C)**. The initial reaction mixture contained 50 mM Tris-HCl (pH 8.0), 5 mM MgCl_2_, 4 mM ATP, 4 mM GTP as well as 500 ng RelS_Cg_ (257 nM) or Rel_Cg_ (118 nM), respectively and was incubated for 4 h at 30°C. The various buffer systems (50 mM each) used to vary the pH value are illustrated by different icons: square: citrate buffer; circle: phosphate buffer; triangle: TRIS buffer; hexagon: ammonia buffer. Mean values and standard deviations shown were calculated from three replicates.

Next, it was interesting to study the dependence of RelS_Cg_ with regard to different reaction parameters. In addition to RelS_Cg_, the enzyme Rel_Cg_ was also characterized to compare the characteristics of monofunctional SAS enzymes and the bifunctional multi-domain RSH proteins. In order to obtain results with high information value on the physiological significance of the enzymes analyzed, the corresponding starting parameters were adapted to natural values as far as possible. Therefore, physiological Mg^2+^ concentrations of 5 mM (Nierhaus, 2014) and the standard *C. glutamicum* growth temperature of 30°C were used. However, preliminary experiments showed low activities for Rel_Cg_ using the optimal *C. glutamicum* cultivation pH of 7. For this reason and due to a lack of data regarding SAS proteins, the characterization was carried out starting from pH optimum values of bifunctional RSH enzymes at pH 8 (Sajish et al., 2007), to increase comparability. Since the values chosen do not correspond to the RelS optimum conditions retrospectively, suboptimal specific activities were achieved in the individual measurements. For RelS_Cg_, a typical bell-shaped curve with a maximum activity in the neutral range of approximately pH 6.5 was found in the analysis of its pH dependency (Figure 6A). The activity extended to a pH range of approximately pH 5.5–pH 9.25. Since only slight differences of the activity values were determined in the various buffer systems used for pH characterization, the activity appears to be largely independent of the buffer-active components phosphate, citrate, or ammonium. In contrast, a distinctly different pH dependency was found for the bifunctional RSH enzyme Rel_Cg_. The enzyme had no measurable pyrophosphokinase activity up to a pH value of 6.25. At higher pH values, an increase in the pppGpp synthesis activity occurred, which reached a maximum value from pH 9 on and still had its full activity at pH 10.

The temperature optimum of RelS was 15°C and at the standard cultivation temperature of 30°C, its activity was only about 70% of the maximum value (Figure 6B). In contrast, a typical exponential increase in activity up to a maximum value at a temperature of 40°C was observed for Rel_Cg_. Higher temperatures led to a drastic reduction in pppGpp production. Both enzymes had very low activities at temperatures above 50°C.

The presence of Mg^2+^ ions turned out to be essential for the pyrophosphokinase activity of both RelS_Cg_ and Rel_Cg_, since no activity could be detected without this divalent cation. Under the conditions applied, almost identical pyrophosphokinase activities in the form of saturation curves were observed for both enzymes up to a MgCl_2_ concentration of 10 mM (Figure 6C). At higher MgCl_2_ concentrations, however, significant differences in activity were observed. For RelS_Cg_, an extreme increase in pppGpp synthetase activity was found in the form of a further saturation curve. At a MgCl_2_ concentration of 50 mM, a maximum pyrophosphokinase activity of 1.375 μmol min^−1^ mg^−1^ was achieved, which was more than 10 times as high as the value reached for 10 mM MgCl_2_. By contrast, the activity of Rel_Cg_ decreased continuously using MgCl_2_ concentrations above 10 mM and was only 0.047 μmol min^−1^ mg^−1^ at 50 mM MgCl_2_.

## Discussion

Although it was long believed that the system of stringent response of the actinobacterial model organism *C. glutamicum* was fully understood, the results presented here show a new, more complex picture of the (p)ppGpp metabolism. Due to their sequence similarity to the catalytically active domains of already analyzed RSH proteins, two possible monofunctional (p)ppGpp synthetases were identified in the genome of the *C. glutamicum* in addition to the already known bifunctional enzyme Rel_Cg_. Such small SASs have been identified by bioinformatic investigations in the genomes of numerous species from almost all prokaryotic phyla (Atkinson et al., 2011), and the functionalities of some of their families have been confirmed for a few model organisms (Lemos et al., 2007; Nanamiya et al., 2008).

Bioinformatic analyses identified the *C. glutamicum* ORFs *cg1330* and *cg2324* to encode putative SAS (small alarmone synthetase) proteins. The protein encoded by *cg1330* was classified as RelP and the protein encoded by *cg2324* was classified as actRel and is named RelS in this study. It was interesting to note that two variants of the *relP* gene exist in different *C. glutamicum* genome sequences. While many *C. glutamicum* strains have a supposed full-length version for the gene, the type strain and its derivatives showed a deletion in its 3′-part, leading to RelP variant which is shortened by 41 aa at its C-terminus and also has an alternative 11 aa C-terminal end sequence. Since this region contains amino acid residues highly conserved in RelP proteins, the shorter version might not be a functional enzyme. Further doubts on the physiological significance of this gene are raised by the fact that the *relP* gene is not conserved in corynebacterial genomes but only present in *C. glutamicum* strains.

The RelP subgroup generally represents an SAS form strongly conserved in Firmicutes, whose activity has already been demonstrated for several species, such as *Streptococcus mutans* and *Bacillus subtilis* (Lemos et al., 2007; Nanamiya et al., 2008). For the well-characterized SAS enzyme YjbM from *B. subtilis*, the formation of tetramer structures and associated regulatory effects by pppGpp were found under physiological conditions (Steinchen et al., 2015). According to sequence alignments performed by Steinchen et al. it is probable that the representatives of the RelP subclass are also active as tetramers. The shortening of the RelP protein of *C. glutamicum* ATCC13032 could have a massive impact on such quaternary structures. A loss of corresponding protein regions, relevant to the formation of the correct tetramer form, might explain the lack of activity. Interestingly, in the complementation experiments carried out, no *in vivo* activity could be detected for the apparent full-length RelP variant RelP_Cg_. Since the gene does not occur in any other species of the family Corynebacteriaceae and the strain *C. glutamicum* ATCC13032 has been able to establish a deletion with a strong effect on the amino acid sequence, there is some evidence that *relP*_Cg_ is a horizontally received sequence whose expression product is not part of the stringent response system in *C. glutamicum*. Based on the *in vitro* characterization which found both, kinase and hydrolase activity in respect to the 5′-group of the nucleotide substrates used, the enzyme RelP^*^_Cg_ possibly plays a role in the primary nucleotide metabolism of *C. glutamicum*.

For the second protein RelS_Cg_, which is encoded by ORF *cg2324*, the bioinformatic classification as pyrophosphokinase could be confirmed. In the course of the characterization by means of complementation of the (p)ppGpp^0^ mutant *E. coli* Δ*relA*Δ*spoT*, however, there were clear differences to the RSH enzyme Rel_Cg_. Different inducer concentration dependencies indicate activity differences between the Rel_Cg_ and RelS_Cg_ enzymes.

By growth analysis of different *C. glutamicum* deletion mutants, a functional relationship between RelS_Cg_ and the bifunctional enzyme Rel_Cg_ could be elucidated. The deletion of *rel*_Cg_ resulted in a marked reduction in cell growth in the course of the cultivation in minimal medium, leading to a stationary phase at an OD_600_ of 4–5. Since this effect could be compensated by the additional deletion of *relS*_Cg_, the expression of this gene without the presence of Rel_Cg_ appears to represent a significant problem for *C. glutamicum*. On the basis of the pyrophosphokinase activity determined for RelS_Cg_ and the absence of a (p)ppGpp hydrolase domain in its protein sequence, it can be assumed that the cells of strain CR099 Δ*rel* enter stationary state due to the lack of Rel_Cg_ hydrolase activity and the thereby increased (p)ppGpp pool. The growth deficiency of CR099 Δ*rel* would thus correspond to the reduction of cell growth in the context of the stringent control in which a high production of the messenger (p)ppGpp is triggered by various stresses, such as nutrient deficiencies. An equivalent functional relationship between a bifunctional RSH enzyme and monofunctional SAS proteins has already been observed for different organisms. For *B. subtilis*, the negatively influenced growth characteristics of the RSH deletion mutant *B. subtilis* Δ*relA* could also be largely compensated by the additional deletion of the SAS genes *yjbM* and *ywaC* (Nanamiya et al., 2008). In *S. mutans*, the overexpression of the SAS gene *relP* led to a reduced growth rate exclusively in a strain with deletion of the RSH gene *relA*, whereas the parental strain was not influenced (Lemos et al., 2007). Both results also indicate the negative effects of the missing hydrolase activity in Δ*rel* or Δ*relA* strains in interrelationship with the (p)ppGpp production by various SAS enzymes. Interestingly, a putative RSH protein was bioinformatically identified in the genome of *C. glutamicum* with the gene *cg1485* (Atkinson et al., 2011). Since a corresponding enzyme is not present in the strains examined so far, the functional relationships within the (p)ppGpp metabolism of *C. glutamicum* could be even more complex and therefore further investigations are needed.

It is interesting to note that the deletion of both enzymes having (p)ppGpp synthetase activity leads to a slightly delayed but otherwise identical growth characteristic in CGXII minimal medium as compared to the parental strain. In contrast to *E. coli, C. glutamicum* appears to be not dependent on a (p)ppGpp basal level during cultivation in minimal medium. Since the triple deletion mutant CR099 Δ*rel*Δ*relP*Δ*relS* grew identically in minimal medium, RelP^*^_Cg_ can be excluded as a possible source of (p)ppGpp. A carryover of complex constituents from the preculture medium can likewise be largely excluded, since the cells were washed intensively with minimal medium before inoculation.

The growth phenotype of *C. glutamicum* CR099 Δ*rel* obtained in this study differs significantly from earlier studies of a *C. glutamicum* Δ*rel* strain, performed by Tauch et al. (2001). For this strain a considerable growth defect with a very long lag-phase was observed during the cultivation in minimal medium, which could be complemented by the addition of serine and histidine. Since the two parental strains CR099 and RES167 are different only with regard to IS elements and prophages, an effect of the parent background is unlikely. Furthermore, both strains have different deletions of the gene *cg1861*. While a complete start-stop deletion was carried out in the current study, only an area of approximately 400 bp was deleted for constructing RES167 Δ*rel*. However, since the deletion is located in the N-terminal region and there is a frameshift leading to the formation of an alternative stop codon, only a 140 aa fragment of the hydrolase domain can be expressed. As a result, both strains are essentially comparable to one another and the differences in the growth characteristics determined are probably attributable to different cultivation conditions. During the cultivation of RES167 Δ*rel*, the main cultures were inoculated with very small cells titers, which can have a negative effect on the initial growth of *C. glutamicum*. Taking this into account the cultivation of Tauch et al. (2001) could have been terminated too early because the strain showed significant OD increases in the last data points of the measurement series. Furthermore, unrecognized changes within the RES167 genome can not be ruled out, since overall genome sequencing was not yet available at the time of strain construction.

The *in vitro* characterization of RelS_Cg_ revealed interesting properties for the substrate concentration dependency as well as the dependence on further reaction parameters. With respect to the two substrates GDP and GTP, substrate excess inhibition could be determined in high concentration ranges of 25–50 mM, which has not yet been observed for other SAS enzymes. However, the biological relevance of this inhibitory effect is extremely low since the necessary concentrations for it to occur are unlikely under physiological conditions. For example, the intracellular nucleotide concentrations in exponentially growing *E. coli* cells were determined to be in the range from 0.024 to 5 mM (GTP: 4.9 mM, GDP: 0.68 mM, GMP: 0.024 mM) (Bennett et al., 2009). Compared to the RelQ homolog of *B. subtilis*, the conversion of GDP is significantly slower since a maximal activity of 6.6 μmol min^−1^ mg^−1^ was determined for this SAS protein (Steinchen et al., 2015). In contrast to RelS_Cg_, RelQ_Bs_ has a lower maximum activity for GTP. With 2 μmol min^−1^ mg^−1^, it reaches only about 70% of the value determined for RelS_Cg_. The RelS K_m_-values of 4.1 mM for GTP and 6.1 mM for GDP also differ markedly from the values determined for other small SASs. The enzyme MS_RHII-RSD from *M. smegmatis*, likewise assigned to the actRel subgroup, has a K_m_ value of 0.9837 mM for GDP and 0.96 for GTP (Murdeshwar and Chatterji, 2012). RelQ from *B. subtilis* also achieves half-maximal activity at significantly lower substrate concentrations of 1.7 mM (GDP) and 1.2 mM (GTP) (Steinchen et al., 2015). RelS_Cg_ thus has a comparatively low affinity for the substrates GDP and GTP.

An untypical optimum value of 15°C was determined during the analysis of RelS_Cg_ with respect to different temperatures. In contrast, a completely different temperature dependence of the pyrophosphokinase activity was observed for the bifunctional RSH enzyme Rel_Cg_. The (p)ppGpp synthetase activity increased exponentially up to a temperature of 40°C and dropped at even higher temperatures, probably due to denaturation of the protein. Since *C. glutamicum* is a soil bacterium, the unusual temperature optimum of RelS_Cg_ may be an adaptation to temperatures in its natural habitat. However, since the activity maximum of this SAS enzyme is clearly below the optimum growth temperature, the result is an indication for a possible association of RelS_Cg_-induced (p)ppGpp production in response to low temperatures. In order to demonstrate such participation in the cold shock response, the involvement of RelS_Cg_ must be verified by appropriate *in vivo* measurements. Another unusual behavior of the RelS_Cg_ activity is based on its MgCl_2_ concentration dependency. The fundamental Mg^2+^ dependence of the pyrophosphokinase reaction, which was found both in RelS_Cg_ and Rel_Cg_, indicates an essential importance in the reaction mechanism and has already been demonstrated for bifunctional RSH proteins (Sajish et al., 2007). In contrast to RelS_Cg_, RSH proteins, such as the enzyme Rel_Mtb_ of *M. tuberculosis* (Sajish et al., 2007) show a marked reduction in enzyme activity with increasing MgCl_2_ concentrations above 5 mM. Based on this behavior, Sajish et al. proposed a single metal ion mechanism for such proteins. For “bifunctional” Rel enzymes which, like RelA from *E. coli*, have exclusively synthetase activity and are also referred to as monofunctional in the corresponding study, there is no decrease in the enzyme activity as a function of the Mg^2+^ concentration. Based on a protein sequence analysis within the bifunctional Rel enzymes, it could be shown that this class of “bifunctional” RelA-like Rel proteins have a dual metal ion mechanism by changing the motif RFKD, to EFDD (Sajish et al., 2007, 2009). As a result of this change, no reduction in the activity takes place with increasing Mg^2+^ concentrations. The values determined for RelS_Cg_, which also show no characteristic reduction of the enzyme activity at high concentrations of Mg^2+^, thus suggest a dual metal ion mechanism. However, since the corresponding amino acid motifs are not conserved in RelS_Cg_, the exact reaction mechanism must be further investigated with the help of additional methods, such as crystal structure analysis or hydrogen–deuterium exchange (HDX) mass spectrometry (Steinchen et al., 2015). In contrast to RelS_Cg_, Rel_Cg_ can be clearly assigned to the RSH proteins with single metal ion mechanism. The protein has both the characteristic Mg^2+^ dependency and the conserved RFKD sequence motif (Sajish et al., 2007). The pH dependencies of the respective pyrophosphokinase activities also show considerable differences between the two enzymes, thus also pointing to different reaction mechanisms. At the pH optimum of 6.5 of RelS_Cg_, Rel_Cg_ shows only 20% of its maximal activity. In the basic pH range, however, the activity of the latter increases significantly while RelS_Cg_ has only a slight (p)ppGpp synthetase activity at pH values above 8.

Interestingly, an Mg^2+^-independent pyrophosphokinase activity was detected for the enzyme MS_RHII-RSD from *M. smegmatis*, which is also assigned to the SAS subgroup actRel (Murdeshwar and Chatterji, 2012). In addition to the (p)ppGpp synthetase domain, the corresponding enzyme has an RNase HII domain and is thus significantly larger than the enzyme RelS_Cg_. Due to the different basic activities as well as the apparently divergent pyrophosphokinase reaction mechanisms, the classification of both proteins to one SAS subgroup is to be assessed as questionable from a functional point of view. Equivalent differences could already be found in the course of the phylogenetic analysis, which illustrated a rather heterogeneous picture for the actRel subgroup, proposed by Atkinson et al. (2011). Especially the RelS homologs of the genus *Corynebacterium* show a clear distance from the other representatives in this context. In order to analyze the interrelations within the subgroup and a possible separation into two or more individual groups, further representatives of this group have to be characterized. In particular, the still unknown biological functions of the different representatives could differ massively due to the additional RNase domain and thus lead to a new classification of the otherwise phylogenetically similar proteins. The exclusive occurrence of RelS homologs in representatives of the genus *Mycobacterium* which are not assigned to the Mtb complex suggests an association of the biological function with environmental factors, such as temperature, salinity, osmotic pressure, etc.

On the basis of the described properties, the analyzed protein RelS_Cg_ is quite suitable for a possible *in vitro* production of the different alarmone species. Especially the strong activity at high substrate concentrations and the extreme increase of the specific product formation rate by the use of unphysiologically high magnesium ion concentrations could enable the establishment of economically interesting production processes of ppGpp or pppGpp.

In the investigation of the product spectra, a reaction to ADP and GTP was detected for all enzymes studied using the substrate combination ATP/GDP. All enzymes thus seem to have a nucleoside diphosphate kinase activity which has not yet been described for RSH or SAS-enzymes. However, this may also be due to the evaluation of previous analyses by means of thin-layer chromatography, which has a low resolution for nucleoside di- and triphosphates among the conditions used. Particularly striking in this context is the strong production of GTP and ADP by Rel_Cg_. The conversion of the resulting GTP in the assay reaction with ATP and GDP could also be responsible for the unexpectedly high pppGpp concentration, although direct phosphorylation of ppGpp to pppGpp is also conceivable. When evaluating *in vitro* characterization, however, it must be noted that the assays carried out are highly artificial systems. The absence of ribosomes, tRNA species and RNA could have a massive effect on the results for Rel_Cg_, since regulatory effects of these cell components are known for different RSH enzymes (Haseltine and Block, 1973; Avarbock et al., 2000). Since a strong reduction of the GTP level was found to be a major functional basis of the stringent response system in Firmicutes (Krásný et al., 2008; Gaca et al., 2015a), an *in vivo* significance of the RSH or SAS-based GTP synthesis in *C. glutamicum* seems unlikely. However, there may also be considerable differences between the stringent response mechanisms of Firmicutes and Actinobacteria.

One of the most interesting results of the performed *in vitro* characterization is the extension of the so far known substrate spectrum of Rel_Cg_ as well as RelS_Cg_ by GMP. The characteristic pyrophosphorylation in the C3 position selectively produces GMP 3'-diphosphates (pGpp). This substance was already found as a product of some SAS and shows regulatory effects similar to the already known alarmones (p)ppGpp in *Enterococcus faecalis* (Gaca et al., 2015b). For this reason, the term (pp)pGpp, originally suggested by Gaca et al. (2015b), might also be used for *C. glutamicum* in the future. Although significantly lower values were determined for the specific pyrophosphokinase activity with respect to GMP in the physiological concentration range, the identification of the reaction product pGpp might represent a considerable extension of the stringent response system in *C. glutamicum*. However, the *in vivo* occurrence of the corresponding reaction or substance for *C. glutamicum* has to be analyzed more precisely. Since slightly different biological functions could already be detected for the two known alarmone species ppGpp and pppGpp, the complexity of the corresponding regulatory processes could be even greater than previously assumed by the involvement of pGpp (Mechold et al., 2013; Steinchen et al., 2015).

## Author contributions

MR, MP, and JK designed, analyzed and interpreted the performed experiments. MP and JK supervised the research. MR and MP performed wet lab experiments. The manuscript was written by MR and revised by MP and JK.

### Conflict of interest statement

The authors declare that the research was conducted in the absence of any commercial or financial relationships that could be construed as a potential conflict of interest.
