# Supplementary material for: Identification and Functional Characterization of Small Alarmone Synthetases in Corynebacterium glutamicum
Source: Front Microbiol. 2017 Aug 21;8:1601. doi: 10.3389/fmicb.2017.01601 (PMC5566576; doi:10.3389/fmicb.2017.01601)
Supplement: Supplementary file 1 [file DataSheet1.DOCX]

Supplementary Material

Identification and Functional Characterization of Small Alarmone Synthetases in *Corynebacterium glutamicum*

**Matthias Ruwe, Jörn Kalinowski, Marcus Persicke***

*** Correspondence:** Corresponding Author: marcusp@cebitec.uni-bielefeld.de

**
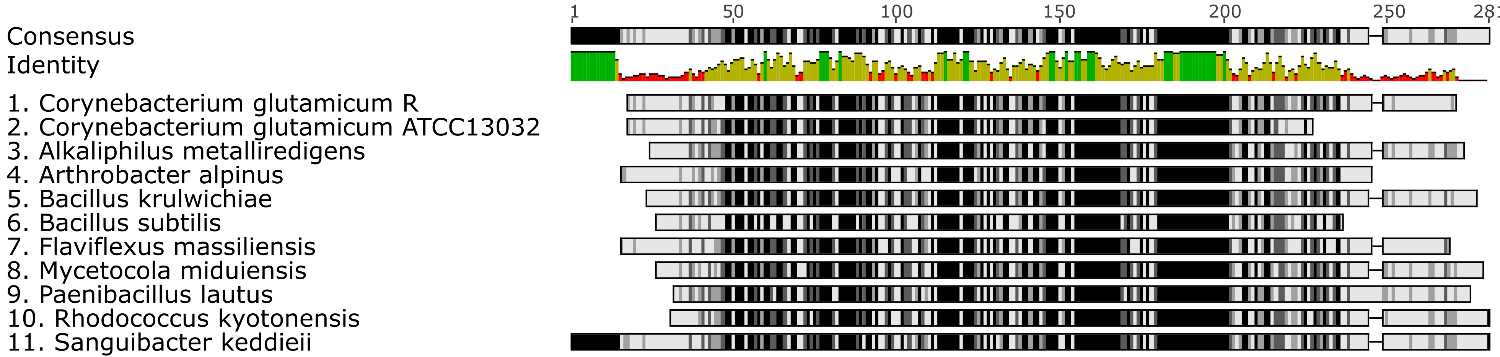
**

**Figure S1.** Multiple alignment of the amino acid sequence of potential RelP_Cg_ homologs selected based on amino acid sequence similarity and represented by the corresponding organisms. Analysis was performed using the program Geneious version 10.1 (Biomatters).


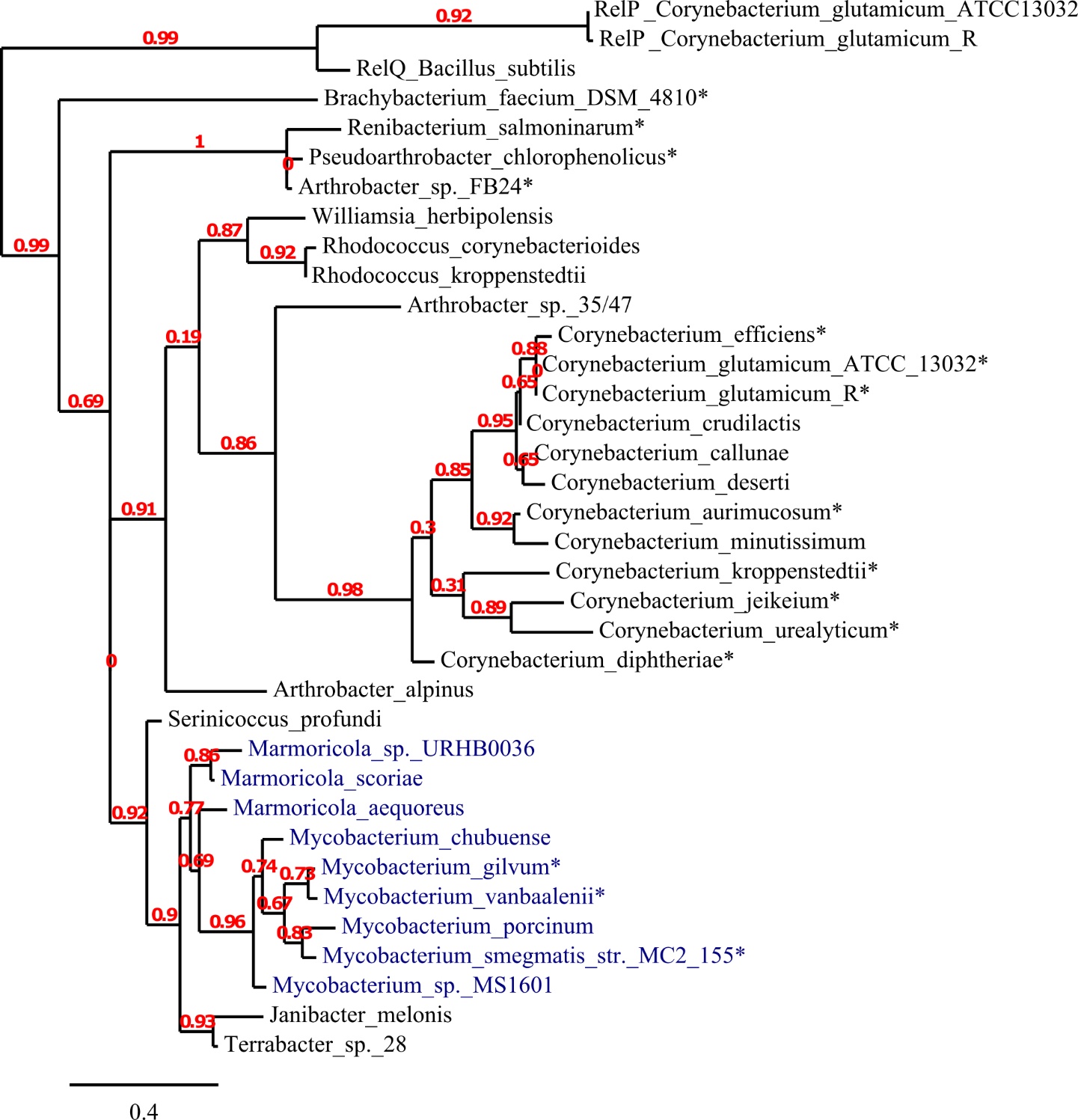


**Figure S2.** Phylogenetic tree of actRel subgroup proteins, represented by the corresponding species. SAS enzymes assigned to the actRel subgroup by Atkinson et al. are denoted by an asterisk (Atkinson et al., 2011). Further proteins represent sequence similarity results for RelS_Cg_ and three proteins from other SAS subgroups. Analysis was performed on the Phylogeny.fr platform, using the MUSCLE (v3.8.31) alignment tool, curation with Gblocks (v0.91b), phylogenetic tree construction with the PhyML program (v3.1/3.0 aLRT) and visualization by TreeDyn (v198.3) (Dereeper et al., 2008; Dereeper et al., 2010). ActRel variants for which the RNaseH-associated protein domain COG4328 was identified alongside the (p)ppGpp synthetase domain are illustrated in dark blue.


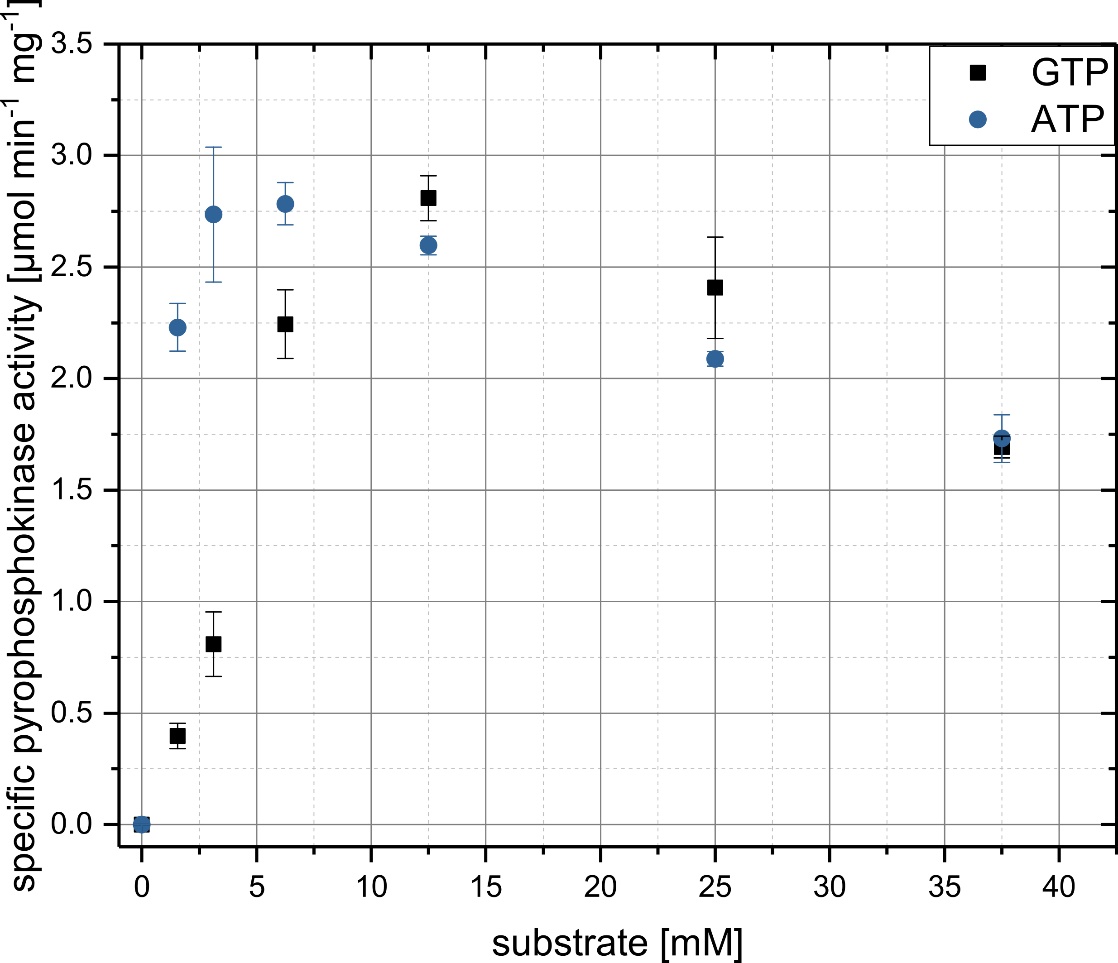


**Figure S3.** Kinetics of RelS_Cg_ with respect to the concentrations of ATP and GTP as substrates. Specific activity was determined for different substrate concentrations by *in vitro* analysis and given in µmol per minute per milligram of RelS_Cg_. Mean values and standard deviations shown were calculated from three replicates.

**Table S1.** Primers used in context of this study. Overlapping sequences, added for Gibson Isothermal Assembly are shown underlined.

| Primer | Sequence | Application |
| --- | --- | --- |
| pBAD_ga1 | ACCTGCAGGCATGCAAGCTT | Construction of pBAD derivates |
| pBAD_ga2 | CATGGTGAATTCCTCCTGCTAGCC |  |
| pBAD_rel1 | TTGGGCTAGCAGGAGGAATTCACCATGAGTCTGGAGCGCAACACAC | Construction of pBAD::*rel*_Cg_ |
| pBAD_rel2 | CAGCCAAGCTTGCATGCCTGCAGGTCTAGCCACCCGAGGTCACTC |  |
| pBAD_relP1 | GGGCTAGCAGGAGGAATTCACCATGTCAGAGAATTTGCCAGCGCCCGAG | Construction of pBAD::*relP*_Cg_ and  pBAD::*relP**_Cg_ |
| pBAD_relP2 | CAGCCAAGCTTGCATGCCTGCAGGTTTAAGTGACTTCACTTCCCCGTGG |  |
| pBAD_relS1 | TTTGGGCTAGCAGGAGGAATTCACCATGTCTGACAACACTCTCTC | Construction of pBAD::*relS*_Cg_ |
| pBAD_relS2 | CAGCCAAGCTTGCATGCCTGCAGGTTTAGTTTGGTGGTTCCACAAG |  |
| pK18_pKrel1 | GACTCCGCATCAGTTCCAATGGATGTGCTGCAAGGCGATT | Construction of pK18*mobsacB*_  *rel*_Cg_ |
| pK18_pKrel2 | CTTTGGAGTACCGGTGATCTTCGCCCTTCCCAACAGTTGC |  |
| pK18_rel1 | GCAACTGTTGGGAAGGGCGAAGATCACCGGTACTCCAAAG |  |
| pK18_rel2 | GAAAGAAAGGCGGCAGGAAAAGGCCTTTAGATTGTGAAAA |  |
| pK18_rel3 | TTTTCACAATCTAAAGGCCTTTTCCTGCCGCCTTTCTTTC |  |
| pK18_re4 | AATCGCCTTGCAGCACATCCATTGGAACTGATGCGGAGTC |  |
| pK18_ga1 | CTGCAAGGCGATTAAGTTGG | Construction of pK18*mobsacB* derivates |
| pK18_ga2 | CTGGCGTAATAGCGAAGAGG |  |
| pK18_relP1 | TCGGTGCGGGCCTCTTCGCTATTACGCCAGTTGGCAGCTGCTGATATCGG | Construction of pK18*mobsacB*_  *relP**_Cg_ |
| pK18_relP2 | TTCACTTCCCCGTGGATTCTTTCTCTCGGCGTCCAGGAGATT |  |
| pK18_relP3 | AATCTCCTGGACGCCGAGAGAAAGAATCCACGGGGAAGTGAA |  |
| pK18_relP4 | CTGGCGTTACCCAACTTAATCGCCTTGCAGCGTCGACTCCCCTCTAAAAG |  |
| pK18_relS1 | TCGGTGCGGGCCTCTTCGCTATTACGCCAGGACGCAGGTTAATTCGGTAG | Construction of pK18*mobsacB*_  *relS*_Cg_ |
| pK18_relS2 | TTTGGCGTTGAGTGGTTGGGGATGGGCACGTCGGAATTCG |  |
| pK18_relS3 | CGAATTCCGACGTGCCCATCCCCAACCACTCAACGCCAAA |  |
| pK18_relS4 | CTGGCGTTACCCAACTTAATCGCCTTGCAGACCGAACAATGGAGCATCTG |  |
| pTXB1_ga1 | ATGTATATCTCCTTCTTAAAGTTAAA | Construction of pTBX2 derivates |
| pTXB1_ga2 | TGCATCACGGGAGATGCACTAGTTG |  |
| pTXB1_relP1 | ACTTTAAGAAGGAGATATACATATGTCAGAGAATTTGCCAGCGCCCGAG | Construction of pTXB1::*relP**_Cg_ |
| pTXB1_relP2 | CAACTAGTGCATCTCCCGTGATGCAAGTGACTTCACTTCCCCGTGGATTC |  |
| pTXB1_relS1 | ACTTTAAGAAGGAGATATACATATGTCTGACAACACTCTCTCCCAATTTG | Construction of pTXB1::*relS**_Cg_ |
| pTXB1_relS2 | CAACTAGTGCATCTCCCGTGATGCAGTTTGGTGGTTCCACAAGATG |  |
| pTXB1_rel1 | GTTTAACTTTAAGAAGGAGATATACATATGAGTCTGGAGCGCAACACAC | Construction of pTXB1::*rel*_Cg_ |
| pTXB1_rel2 | CAACTAGTGCATCTCCCGTGATGCAGCCACCCGAGGTCACTCGGTAG |  |
| relA_redET1 | TAGTTGCGATTTGCCGATTTCGGCAGGTCTGGTCCCTAAAGGAGAGGACGAACCCTCACTAAAGGGCGGC | Deletion of *relA* from *E. coli* |
| relA_redET2 | GTAGATACAGTATATATCAATCTACATTGTAGATACGAGCAAATTTCGGCCGACTCACTATAGGGCTCGA |  |
| spoT_redET1 | CCGTTACCGCTATTGCTGAAGGTCGTCGTTAATCACAAAGCGGGTCGCCCAACCCTCACTAAAGGGCGGC | Deletion of *spoT* from *E. coli* |
| spoT_redET2 | CTGGCGAGCATTTCGCAGATGCGTGCATAACGTGTTGGGTTCATAAAACACGACTCACTATAGGGCTCGA |  |

Supplementary References

Atkinson, G. C., Tenson, T., and Hauryliuk, V. (2011). The RelA/SpoT homolog (RSH) superfamily: distribution and functional evolution of ppGpp synthetases and hydrolases across the tree of life. *PloS one* 6, e23479. doi: 10.1371/journal.pone.0023479

Dereeper, A., Audic, S., Claverie, J.-M., and Blanc, G. (2010). BLAST-EXPLORER helps you building datasets for phylogenetic analysis. *BMC evolutionary biology* 10, 8. doi: 10.1186/1471-2148-10-8

Dereeper, A., Guignon, V., Blanc, G., Audic, S., Buffet, S., Chevenet, F., et al. (2008). Phylogeny.fr: robust phylogenetic analysis for the non-specialist. *Nucleic acids research* 36, W465-9. doi: 10.1093/nar/gkn180
